# Editorial: Strategies for Mitigating the Environmental Impacts of Pig and Poultry Production

**DOI:** 10.3389/fvets.2022.892340

**Published:** 2022-08-17

**Authors:** Ines Andretta, Aline Remus, Florence Garcia-Launay, Luciano Hauschild, Marcos Kipper

**Affiliations:** ^1^Animal Science Department, Universidade Federal do Rio Grande do Sul, Porto Alegre, Brazil; ^2^Agriculture and Agri-Food Canada, Sherbrooke, QC, Canada; ^3^PEGASE, INRAE, Institut Agro, Saint-Gilles, France; ^4^Animal Science Department, São Paulo State University, Jaboticabal, Brazil; ^5^Elanco Animal Health, São Paulo, Brazil

**Keywords:** broiler, environment, life-cycle assessment, livestock, swine

Pig and poultry production are crucial agriculture-based industries in many countries. These production systems are frontlines in the fight against food insecurity. However, these livestock sectors also contribute to the excretion of potential pollutant substances to the environment, like nitrogen and phosphorus. Furthermore, cereals grains used in poultry and pig feeding are also related to land use and many emissions from the cultivation to the processing steps.

In recent years, the poultry and pig industries have advanced in reducing their impacts on the environment. However, there is still much room for improvement, and scientists can contribute to greener animal production by proposing and testing solutions that make these production systems more environmental-friendly. This Research Topic presented scientific evidence that is possible and viable to mitigate the environmental impact of poultry and pig production systems using currently available nutritional tools.

The feed has a major contribution to the environmental impacts of pig and broiler production. However, it should be noted that animal nutrition research has a critical role in attenuating this impact. The use of nutritional tools is one of the main insights provided by Andretta et al. after performing a systematic review of original studies that estimated the environmental impacts associated with both pig (55 studies) and poultry productions (30 studies). The study's conclusion supports the hypothesis that novel feeding techniques may be necessary to mitigate the environmental footprint of both production chains.

One of the most promising options to mitigate environmental impacts is to optimize nutrient-use efficiency by applying precision feeding techniques, as highlighted by Pomar et al.. The potential of novel diet formulation strategies as tools to mitigate the carbon footprint was also described in this paper, as well as the several limitations of standard formulation methods in the context of conventional and precision feeding systems. In agreement with this premise, innovative formulation methodologies that incorporate the environmental impacts of feed ingredients were described and validated by de Quelen et al. as efficient ways to reduce the environmental impacts of pig production without compromising animal performance.

In addition to this innovative formulation method, consistent information on the animal nutrient requirements and the feedstuff characteristics are non-negotiable items when proposing a precision feeding program. The methods available to accurately evaluate the nutritional values of feed ingredients and to assess phosphorus requirements were reviewed by Lautrou et al.. Undoubtedly, a better understanding of the nutritional requirements is of utmost importance for mitigating pollutant excretion. To accomplish this, an alternative modeling framework that incorporated uncertain traits of individual pigs in a precision feeding modeling framework was presented by Misiura et al.. This data-driven approach can improve the estimation of individual nutrient requirements and, therefore, the economic and environmental sustainability of pig production systems.

Still focusing on mitigation options related to the feed formulas, the beneficial effects of supplementing feed-grade amino acids on the environmental impacts of broiler and pig production were reviewed by Cappelaere et al.. The current knowledge on using low-crude protein diets was summarized, and factual research information was provided to quantify direct (feed production) and indirect (emissions from manure) impacts. In addition, Hickmann et al. provided several results indicating the potential of using feed additives as eco-friendly strategies during formulation. The study was developed focusing on β-mannanase supplementation, which reduced the amount of soybean oil used in feed formulas and, consequently, mitigated the environmental impacts of pig and poultry feeding programs. Both papers highlighted the importance of choosing feed ingredients considering not only performance or economic criteria but also the environmental standpoint.

In fact, growth performance and environmental aspects cannot be separated. The relationships between different performance selection traits and environmental impacts were evaluated by Monteiro et al. in individual growing pigs. This study concluded that genetic selection to improve feed conversion ratio is the best option to benefit both performance and environmental impacts. The authors also suggested using a similar approach on actual data (e.g., information collected by genetic companies). Another important insight from this study is that improving performance can be a way to improve environmental sustainability too. Indeed, the findings presented by Chen et al. revealed a significant correlation between sow gut microbiota and litter size, providing another piece of evidence that several responses are connected in animal science and that an overall assessment should be preferred instead of focusing on isolated impacts.

Evaluating the overall aspects of a given system is not an easy task, but protocols or assessment routines can help. Thus, a protocol to assess the energy performance of broiler facilities was developed and presented by Baxevanou et al.. After applying the protocol in production units with different technology levels (study-case), the authors also proposed energy-saving measures that can mitigate the environmental footprint of broiler farms, which included proper insulation. In addition, efficient climatization is vital to save energy and prevent physiological and metabolic implications of heat stress on broilers. These effects were reviewed by Nawaz et al., who also suggested strategies to improve broiler production in a warming world.

All the studies presented in this Research Topic can be seen as attempts to improve the way pigs and poultry are raised nowadays. Facing challenges of modernization, farmers and food producers have become more efficient over the last decades; however, the systems' environmental sustainability still needs improvements. We do believe the more the production chains are studied, the more effective the actions will be to move toward a more resource-efficient and sustainable food production system. This aspect is particularly crucial because the growing demand for food worldwide must be met at an affordable cost without compromising environmental integrity. Access to food is a fundamental human right. This Research Topic demonstrated that scientists are working worldwide to make food available with as little impact as possible to meet current demands and ensure the same right for the next generations.

## Author Contributions

IA and MK wrote the first draft of the manuscript. All authors contributed to manuscript revision and approved the submitted version.

## Conflict of Interest

AR was employed by Agriculture and Agri-Food Canada, Sherbrooke, QC, Canada. MK was employed by Elanco Animal Health, São Paulo, Brazil. The remaining authors declare that the research was conducted in the absence of any commercial or financial relationships that could be construed as a potential conflict of interest.

## Publisher's Note

All claims expressed in this article are solely those of the authors and do not necessarily represent those of their affiliated organizations, or those of the publisher, the editors and the reviewers. Any product that may be evaluated in this article, or claim that may be made by its manufacturer, is not guaranteed or endorsed by the publisher.

